# Supplementary material for: Customizable FDM-based zebrafish larva mold for live imaging
Source: Biol Open. 2026 Apr 24;15(4):bio062406. doi: 10.1242/bio.062406 (PMC13148467; doi:10.1242/bio.062406)
Supplement: Supplementary information [file biolopen-15-062406-s1.pdf]

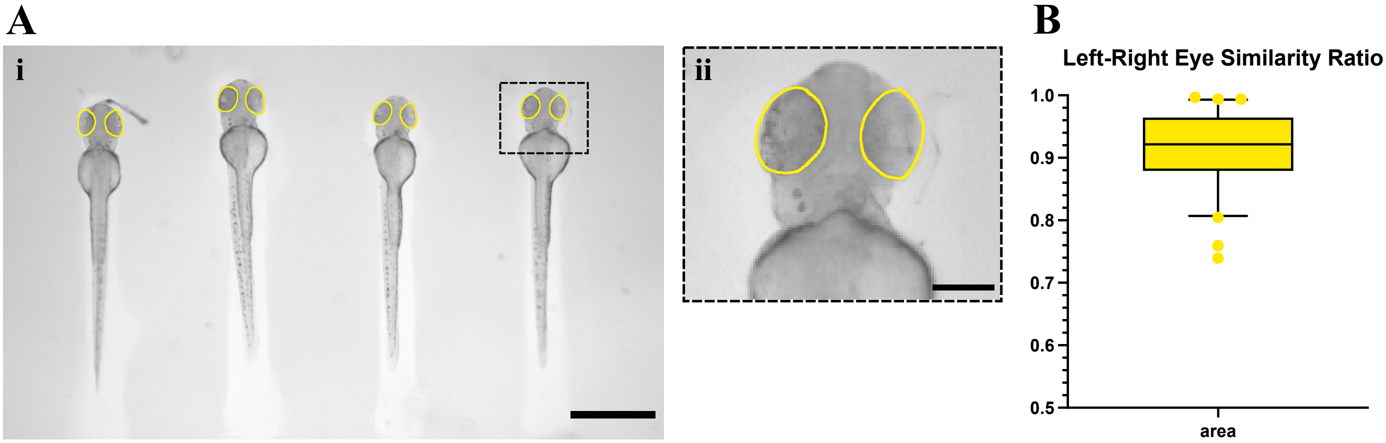

Fig. S1. Quantitative assessment of larvae positioning reproducibility using the left-right eye similarity ratio. A) Representative brightfield images of 52 hpf larvae mounted in the FDM-based molds. (i) Left and right eyes are outlined (yellow) to illustrate the measurements used for quantification; the black-dotted box indicates the region shown at higher magnification (ii). Scale bars: 10 mm and 2 mm, respectively. **B**) Distribution of left-right similarity ratio scores for eye area across all mounted larvae (n = 71). Similarity was calculated as S = min(L, R)/max(L, R), where S = 1 indicates identical values for the left and right eyes. Results are displayed as a box plot showing the median with the center line. The lower and upper edges of the box represent the 25th and 75th percentiles. The whiskers indicate the 5th and 95th percentiles, with individual dots representing data points outside this range.

Table S1. Assessment of larvae viability when mounted in the molds. Viability of larvae mounted at 52 hpf in five zebrafish molds (A= small (ø = 13 mm); B-E= large (ø = 20 mm) was assessed 24 hours after mounting incubation at 28.5 °C by checking the presence of a heartbeat.

Plate	Larvae (total)	Alive (with heartbeat)	Dead (no heartbeat)	% alive
A ($\varnothing = 13 mm$)	7	7	0	100
B ($\varnothing = 20 mm$)	20	20	0	100
C ($\varnothing = 20 mm$)	13	13	0	100
D ($\varnothing = 20 mm$)	18	18	0	100
E ($\varnothing = 20 mm$)	18	18	0	100

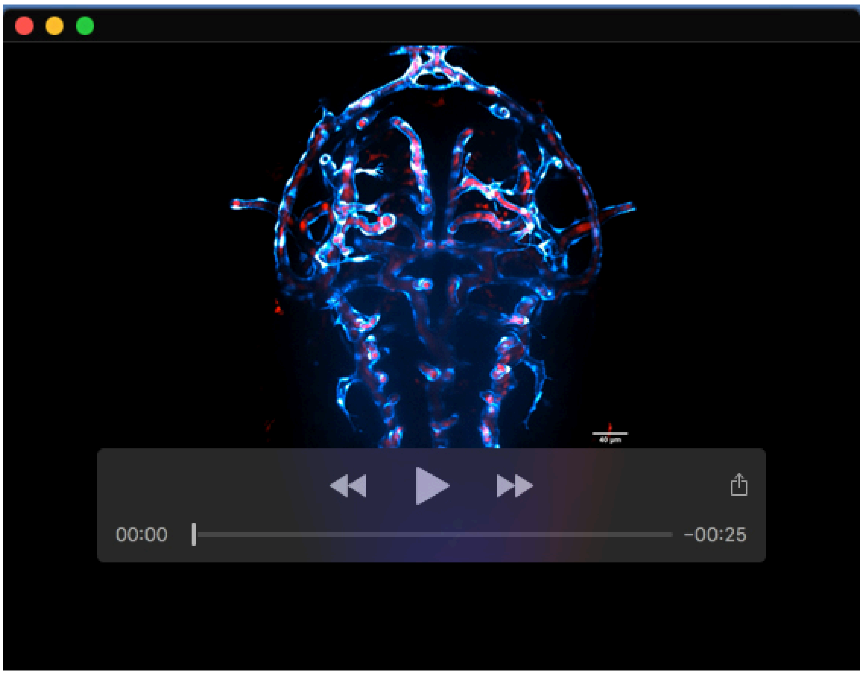

Movie 1. Brain vascularization in the developing brain of a mounted larva.
The development of the brain vasculature and erythrocyte flow in more mature vessels was imaged in larvae expressing both *Tg(fli1a:GFP-CAAX)* (cyan) and *Tg(gata1a:DsRed)* (red) reporters. For parts A and B, the time intervals are 20 and 2 min, and the scale bars are 40 µm and 20 µm, respectively. For part A, acquisition was started at 54 hpf, and for part B, at 68 hpf (using the same larva).

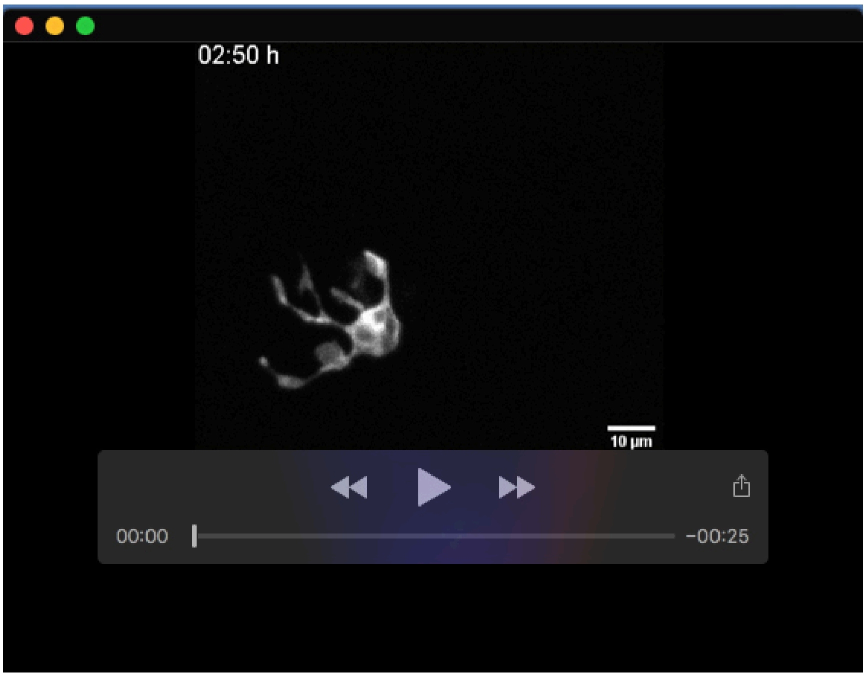

Movie 2. Microglia migration and process extension dynamics in the developing brain of a mounted larva.
Single microglial migration and the elongation and retraction of its processes, labelled by the *Tg(fmpeg1.1:GFP-CAAX)* reporter (grey), were imaged for 12 hours. Time interval: 10 minutes. Acquisition started at 72 hpf. Scale bar 10 µm.

Biology Open • Supplementary information
